# Prediabetes and all-cause mortality in young patients undergoing coronary artery angiography: a multicenter cohort study in China

**DOI:** 10.1186/s12933-023-01776-w

**Published:** 2023-03-01

**Authors:** Yibo He, Hongyu Lu, Yihang Ling, Jin Liu, Sijia Yu, Ziyou Zhou, Tian Chang, Yong Liu, Shiqun Chen, Jiyan Chen

**Affiliations:** 1 Department of Cardiology, Guangdong Cardiovascular Institute, Guangdong Provincial People’s Hospital, Guangdong Academy of Medical Sciences, Guangzhou, 510080 China; 2 Department of Guangdong Provincial Key Laboratory of Coronary Heart Disease Prevention, Guangdong Cardiovascular Institute, Guangdong Provincial People’s Hospital, Guangdong Academy of Medical Sciences, Southern Medical University, Guangzhou, 510080 China; 3 The Second School of Clinical Medicine, Southern Medical University, Guangzhou, 510515 Guangdong China; 4 School of Medicine, South China University of Technology, Guangzhou, 510006 China; 5 Global Health Research Center, Guangdong Provincial People’s Hospital, Guangdong Academy of Medical Science, Southern Medical University, Guangzhou, 510100 China

**Keywords:** Coronary artery angiography, Mortality, Prediabetes, Young adults

## Abstract

**Background:**

The prevalence of prediabetes is increasing in young adults and patients undergoing coronary angiography. However, whether prediabetes is a considerable risk factor for all-cause mortality remains undetermined in young patients undergoing coronary angiography.

**Methods:**

In this study, we retrospectively included 8868 young patients (men aged < 45 years, women aged < 55 years) who underwent coronary angiography (CAG). Patients were categorized as normoglycemic, prediabetes and diabetes according to the HbA1c level or documented history of diabetes. The association of all-cause mortality with diabetes and prediabetes was detected by Cox proportional hazards regression analysis.

**Results:**

A total of 3240 (36.5%) among 8868 young patients receiving CAG were prediabetes and 2218 (25.0%) were diabetes. 728 patients died during a median follow-up of 4.92 years. Compared to the normoglycemic group, prediabetes increased the risk of all-cause mortality in young CAG patients by 24%(adjusted HR: 1.24, 95% CI: 1.04–1.49, p = 0.019) and diabetes increased the risk of all-cause mortality by 46%(adjusted HR:1.46, 95% CI:1.2–1.79, p < 0.001). Subgroup analysis showed that diabetes and prediabetes increased the risk of death mainly in patients without comorbidities.

**Conclusion:**

Prediabetes accounts for more than one-third of the young adults undergoing CAG and was associated with an increased risk of all-cause mortality, active prevention strategy should be considered for these patients.

## Introduction

Prediabetes is defined as the intermediate metabolic state between normoglycemia and diabetes mellitus. According to the American Diabetes Association (ADA) guidelines, prediabetes is defined as the level of HbA1c ranged from 38.8 mmol/mol (5.7%) to 47.5 mmol/mol (6.4%) for patients without known diabetes [1]. Previous studies have demonstrated the current prevalence of prediabetes is elevated, especially in the young population [2, 3]. In a survey of the Chinese general population, the prevalence of prediabetes in adults aged 30–39 years has reached 29.9% [4]. On the other hand, prediabetes was reported more prevalent in patients undergoing coronary angiography (CAG) than in the general population [5, 6]. Marín also found that prediabetes is common in young patients with ST-elevation myocardial infarction (STEMI). As a conventional method for diagnosis of coronary artery disease (CAD), the number of people undergoing coronary artery angiography is increasing [7], with a notable increase in young adults [8]. However, there is still a lack of research on the prevalence and impact of prediabetes on young patients undergoing CAG in China.

Studies have shown that prediabetes increases the risk of cardiovascular disease (CVD) and kidney disease with increased mortality [9, 10], while reversing to normoglycemia from prediabetes prompted reducing the corresponding risk [11]. Compared with old adults, young adults with abnormal blood glucose are reported with a higher risk of mortality [12]. However, it has also been found that in patients with CAD, prediabetes is not associated with the risk of cardiovascular mortality and all-cause mortality [13]. The relationship between prediabetes and cardiovascular disease and all-cause mortality remains equivocal in young patients.

In this study, we aim to investigate the prevalence and effect of prediabetes on all-cause mortality in a large, multi-center cohort of young patients undergoing coronary angiography in China.

## Methods

### Study population

**Fig. 1:**
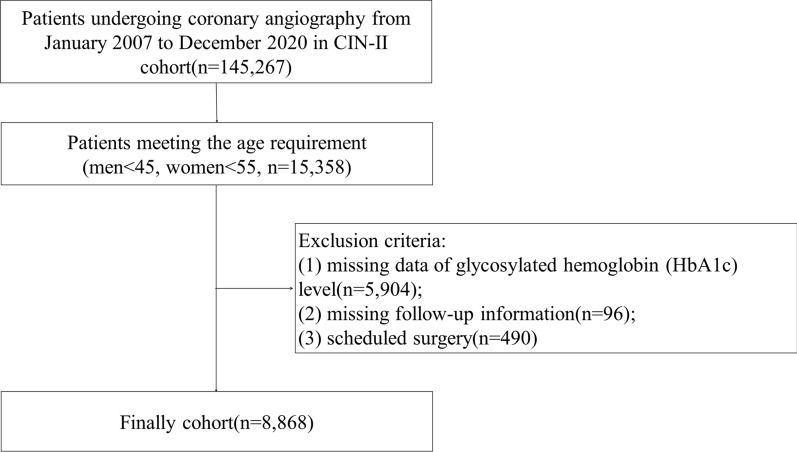
Flowchart of the study

This cohort study analyzed data from the Cardiorenal Improvement II (CIN-II) study, which is a multi-center cohort study with patients enrolled at five large tertiary hospitals (Cardiorenal Improvement II, ClinicalTrials.gov NCT05050877) in China. A total of 145,267 patients undergoing CAG from January 2007 to December 2020 were enrolled. We included patients of young age (men < 45, women < 55; n = 15,358). Patients with missing data on glycosylated hemoglobin (HbA1c) level (n = 5,904), or follow-up information (n = 96), with scheduled cardiac surgery (n = 490) were excluded. Eventually, 8868 patients undergoing CAG were enrolled (Fig. 1). The study was approved by the ethics committee of the participant hospital and complied with the Declaration of Helsinki.

### Data collection

Data was extracted from the electronic clinical management system (ECMS) of each participant hospital. The baseline data comprised the demographic details, medical history, laboratory examination and other clinical information. Patients were subjected to follow-up by trained nurses or assistants after discharge and the follow-up data was obtained by telephone or clinical visits to the patients, otherwise the National Death Registry Database was searched for mortality outcome if necessary.

### Definition and outcome

The ADA’s standards in HbA1c were adopted for the definition of prediabetes and diabetes. Patients with HbA1c lower than 38.8 mmol/mol (5.7%) and no prior diagnosis of all kinds of diabetes were categorized as normoglycemic, with HbA1c ranging from 38.8 mmol/mol (5.7%) to 47.5 mmol/mol (6.4%) and no prior diagnosis of all kinds all diabetes were defined as prediabetes while HbA1c higher than 47.5 mmol/mol (6.5%) or with documented hypoglycemic therapy were defined as diabetes (Type II). The estimated glomerular filtration rate (eGFR) was calculated using the Chronic Kidney Disease Epidemiology Collaboration (CKD-EPI) equation [16], and chronic kidney disease (CKD) was defined as eGFR < 60 mL/min/1.73m^2^ [17]. Congestive heart failure (CHF) was defined as New York Heart Association class > 2 or Killip class > 1. Acute myocardial infarction (AMI), type 2 diabetes mellitus, and hypertension (HT) were defined according to the 10th Revision Codes of the International Classification of Diseases (ICD-10).

The primary outcome was the all-cause mortality which was acquired from the follow-up information recorded by trained staff, the research in the National Death Registry Database was also available for mortality outcome if necessary.

### Statistical analysis

Continuous variables were presented as means (standard deviations [SD]) or median quartiles (IQRs), categorical variables were presented as frequency counts and percentages. Patients' demographic characteristics, medical histories, admission information, and clinical features were listed. One-way analysis of variance (ANOVA) was used to investigate differences between each group. Kaplan–Meier curves were constructed to visually represent time-to-event data, and log-rank tests were employed to evaluate survival across the groups. Covariates enrolled in the multivariate model were screened by stepwise cox regression analysis and based on the clinical significance, including age, gender, triglycerides (TG), low-density lipoprotein cholesterol (LDL-C), CKD, CHF, CAD, AMI, percutaneous coronary intervention (PCI), angiotensin-converting enzyme inhibitor/ angiotensin receptor blocker (ACEI/ARB), beta-blocker, calcium channel blocker, uremic acid, estimated glomerular filtration rate, systolic blood pressure and history of smoking. Multiple imputation was performed for the missing data. To avoid the potential collinearity between variables, variance inflation factors (VIF) were calculated. Subgroup analyses were also performed according to different comorbidities including age, CAD, CHF, CKD, AMI, and PCI. P values derived from two-tailed tests, and values < 0.05 were deemed statistically significant. All statistical analyses were performed using R, version 4.0.3 software (R Foundation for Statistical Computing, Vienna, Austria).

## Results

### Baseline characteristics

A total of 8868 young patients (mean age 43.99 ± 6.90 years, 53.6% were men) undergoing CAG were enrolled in the study. 2218 (25.0%) patients were categorized as diabetes while 3240 (36.5%) were prediabetes. There were 4777 (54.4%) patients were diagnosed with CAD, 3608 (40.7%) patients underwent PCI, 3195 (36.4%) with hypertension, 1754 (20.0%) with acute myocardial infarction (AMI), 984 (11.2%) with CHF, and 511 (5.8%) with CKD. Compared to the normoglycemic group, patients with prediabetes and diabetes were older, more likely to be female and have comorbidities such as AMI, CHF, CKD, CAD, and hypertension. Their TG, TC and LDL-C were higher than those with normoglycemic, while the levels of high-density lipoprotein cholesterol (HDL-C) were lower. The details of the clinical baseline characteristics are shown in Table 1.

**Table 1:** Baseline characteristics according to the glycemic status

Characteristic	Metabolic status				
	Overall	Normoglycemia	Prediabetes	Diabetes	P-value
	n = 8868	n = 3410	n = 3240	n = 2218	
Demographic characteristics					
Age(years)	44.0 ± 6.9	42.5 ± 7.2	44.8 ± 6.5	45.0 ± 6.6	< 0.001
Female	4112(46.4%)	1408(41.3%)	1585(48.9%)	1119(50.5%)	< 0.001
Medical history and clinical condition					
Smoke					0.197
None	4533(74.5)	1688(73.4)	1742(74.3)	1103(76.5)	
Current	1316(21.6)	517(22.5)	518(22.1)	281(19.5)	
Past	235(3.9)	94(4.1)	84(3.6)	57(4.0)	
AMI	1754(20.0)	665(19.6)	526(16.5)	563(25.6)	< 0.001
CHF	984(11.2)	311(9.2)	327(10.2)	346(15.7)	< 0.001
CKD	511(5.8)	135(4.0)	159(4.9)	217(9.8)	< 0.001
CAD	4777(54.4)	1685(49.7)	1579(49.4)	1513(68.7)	< 0.001
PCI	3608(40.7)	1230(36.1)	1189(36.7)	1189(53.6)	< 0.001
COPD	11(0.1)	5(0.1)	2(0.1)	4(0.2)	0.428
HT	3195(36.4)	999(29.5)	1109(34.7)	1087(49.4)	< 0.001
AF	338(3.8)	98(2.9)	157(4.9)	83(3.8)	< 0.001
SBP (mmHg)	126.8 ± 18.7	125.6 ± 18.0	126.3 ± 18.5	129.5 ± 19.8	< 0.001
Laboratory examination					
HbA1c (%)	6.2 ± 1.4	5.3 ± 0.3	6.0 ± 0.2	7.8 ± 1.8	< 0.001
Neutrophil(× 10^12^/L)	5.3 ± 2.9	5.1 ± 2.8	5.0 ± 2.7	5.8 ± 3.1	< 0.001
Lymphocyte(× 10^12^/L)	2.1 ± 0.7	2.0 ± 0.7	2.2 ± 0.7	2.2 ± 0.8	< 0.001
Albumin(g/L)	39.2 ± 4.3	39.6 ± 4.2	39.2 ± 4.2	38.7 ± 4.7	< 0.001
TG (mmol/L)	1.9 ± 1. 6	1.6 ± 1.3	1.8 ± 1.3	2.4 ± 2.1	< 0.001
TC (mmol/L)	4.8 ± 1.3	4.7 ± 1.3	4.9 ± 1.3	4.9 ± 1.4	< 0.001
HDL-C(mmol/L)	1.1 ± 0.3	1.1 ± 0.3	1.1 ± 0.3	1.0 ± 0.3	< 0.001
LDL-C(mmol/L)	3. 0 ± 1.1	2.9 ± 1.0	3.1 ± 1.1)	3.0 ± 1.1	< 0.001
eGFR(ml/min/1.73m^2^)	100.4(85.2, 108.8)	101.8(87.3, 109.7)	99.5(85.0, 107.4)	100.4(80.9, 109.4)	< 0.001
Uric acid(μmol/L)	382.6 ± 114.8	374.3 ± 110.3	385.4 ± 111.6	391.6 ± 124.8	< 0.001
Medication during hospitalization					
ACEI	2985(36.0)	1056(34.0)	1062(34.7)	867(40.7)	< 0.001
ARB	1535(18.5)	468(15.1)	544(17.8)	523(24.6)	< 0.001
β-blockers	5617(67.7)	1996(64.3)	1986(64.9)	1635(76.8)	< 0.001
CCB	1986(23.9)	737(23.7)	681(22.3)	568(26.7)	0.001
Statins	6082(73.3)	2164(69.7)	2167(70.8)	1751(82.2)	< 0.001
Aspirin	5534(66.7)	1975(63.6)	1907(62.3)	1652(77.6)	< 0.001
Diuretic	1198(14.4)	355(11.4)	480(15.7)	363(17.1)	< 0.001

*AMI* acute myocardial infarction, *CHF* congestive heart failure, *CKD* chronic kidney disease, *CAD* coronary artery disease, *PCI* percutaneous interventions, *COPD* chronic obstructive pulmonary disease, *HT* hypertension, *AF* atrial fibrillation, *SBP* Systolic blood pressure, *HbA1c* glycosylated hemoglobin, *TG* triglycerides, *TC* total cholesterol, *LDL-C* low-density lipoprotein cholesterol, *HDL-C* High-density lipoprotein cholesterol, *eGFR* estimated glomerular filtration rate, *ACEI* angiotensin-converting enzyme inhibitor, *ARB* angiotensin receptor blocker, *CCB* calcium channel blocker

### Prediabetes and clinical outcomes

**Fig. 2:**
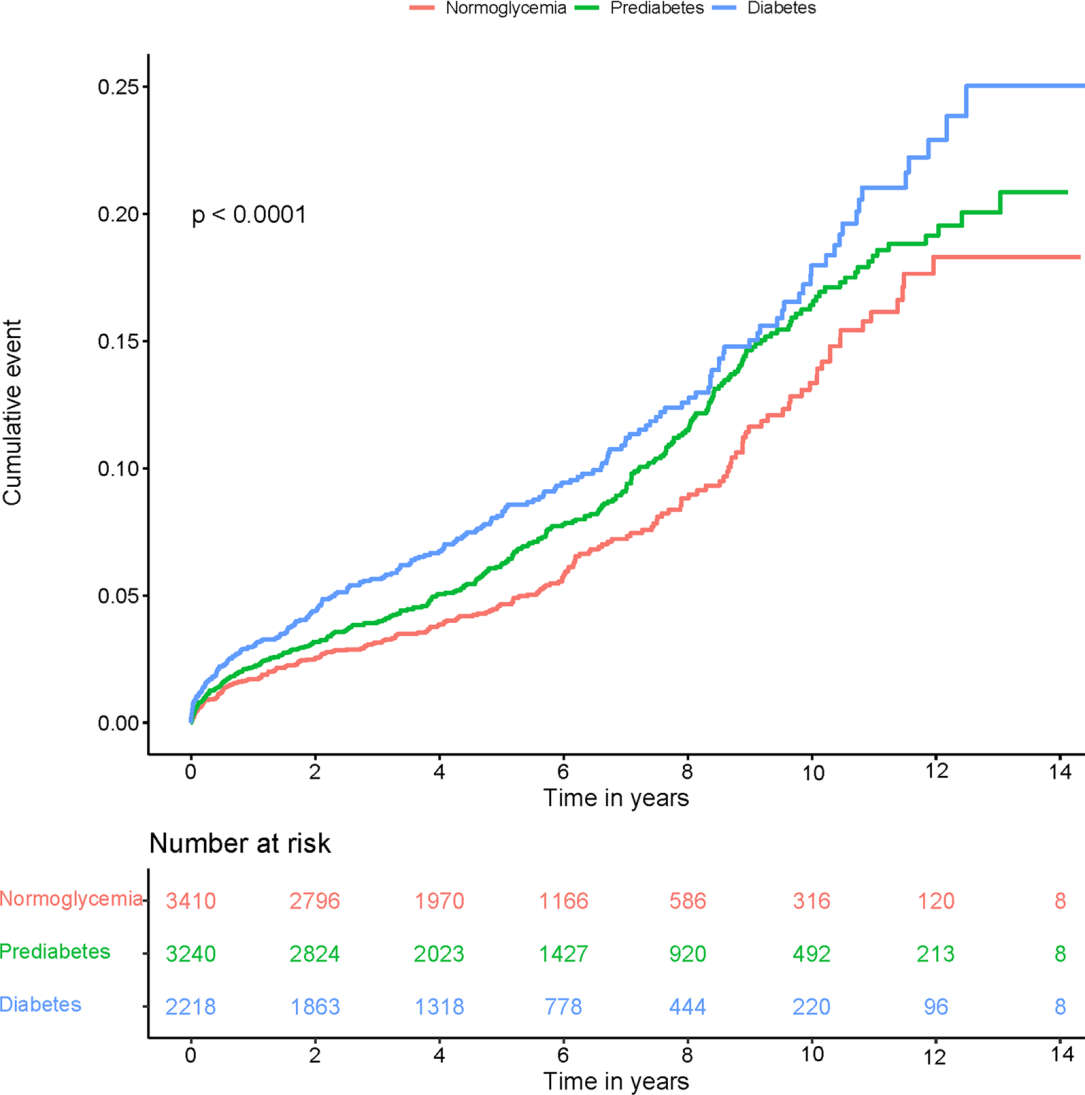
Kaplan–Meier curves of all-cause mortality

**Fig. 3:**
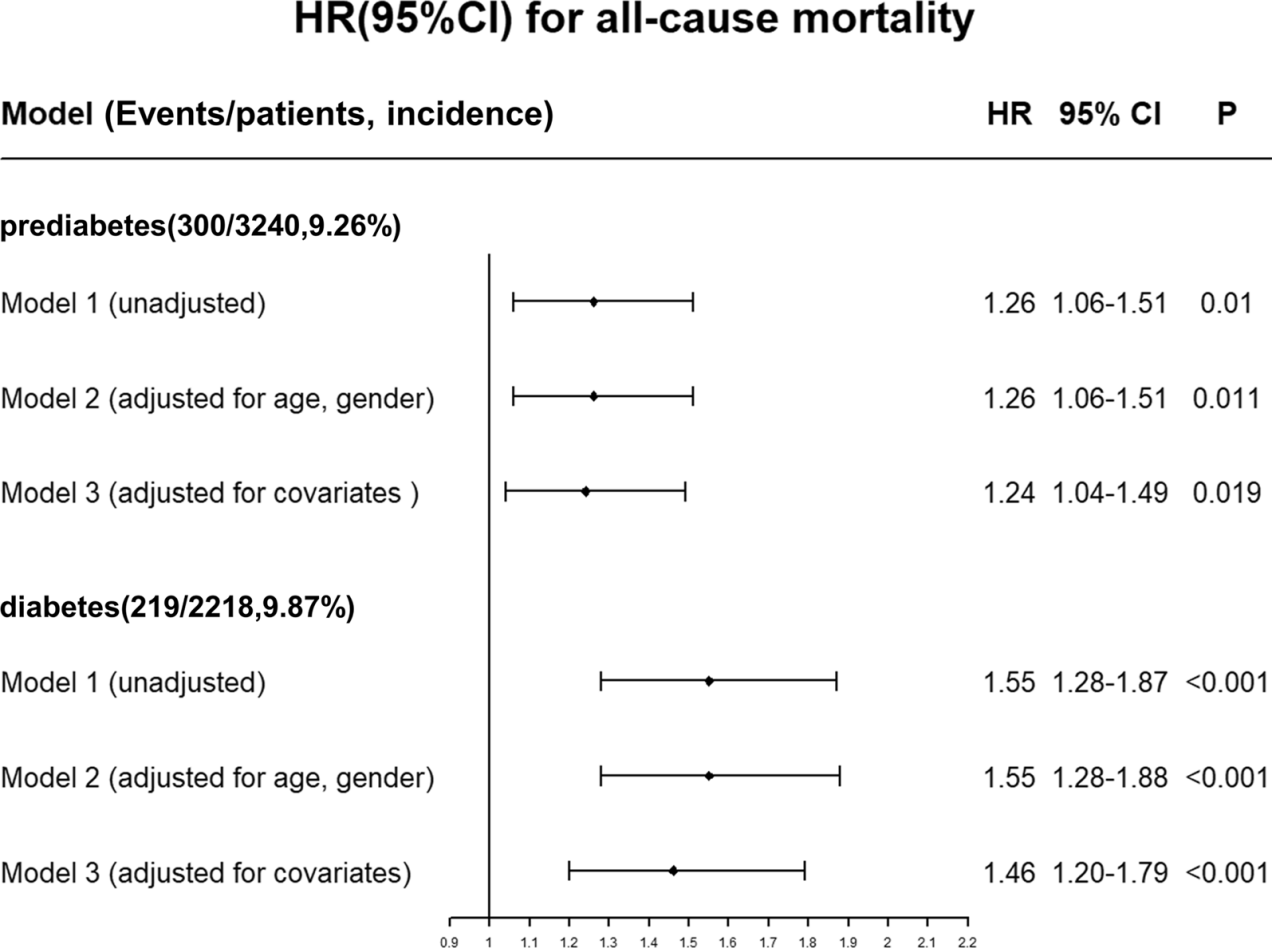
Unadjusted and adjusted HRs and 95% CIs for the primary end point (all-cause mortality) of diabetes and prediabetes Model 1: unadjusted; Model 2: adjusted for age and gender; Model 3: adjusted for age, gender, triglycerides, low-density lipoprotein cholesterol, angiotensin-converting enzyme inhibitor/angiotensin receptor blocker, beta-blocker, calcium channel blocker, uremic acid, estimated glomerular filtration rate, systolic blood pressure, history of smoking, and comorbidities including chronic kidney disease, congestive heart failure, coronary artery disease, acute myocardial infarction, percutaneous coronary intervention

During a mean follow-up of 4.92 years, a total of 728 patients died including 209 in the normoglycemia group, 300 in the prediabetes group and 219 in the diabetes group. The time-to-event curves showed that patients with prediabetes had a increased risk in all-cause mortality compared with normoglycemic patients, and diabetes was associated with a higher risk of mortality (Fig. 2). Cox regression analysis showed that prediabetes and diabetes significantly increased all-cause mortality in young CAG patients by comparison to the normoglycemic group (adjusted HR: 1.24, 95% CI: 1.04–1.49, p = 0.019; adjusted HR:1.46, 95% CI:1.2–1.79, p < 0.001) (Fig. 3). The variance inflation factors showed no significant covariance among each of the incorporated covariates (VIF < 5).

### Subgroup analysis

**Fig. 4:**
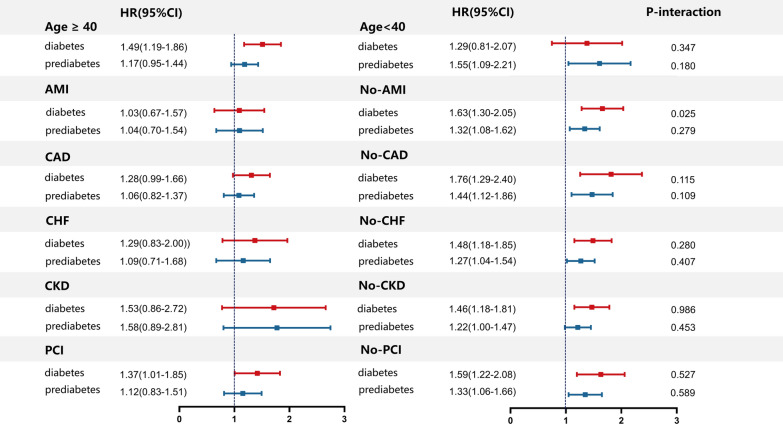
Forest plots of hazard ratio for all-cause mortality in subgroup

In the subgroup analysis, Cox regression analysis showed that diabetes increased the risk of mortality in patients without AMI, CAD, CHF, CKD, age ≥ 40 years old and those with or without receiving PCI at baseline (Fig. 4). On the other hand, prediabetes was associated with the elevated risk of mortality in patients without AMI, CAD, CHF, CKD, PCI and age < 40. However, the interactions between the subgroups regarding the effect of prediabetes and diabetes on mortality were insignificant generally.

## Discussion

In summary, our data showed that prediabetes increased the risk of all-cause mortality in young patients undergoing CAG by 24% and diabetes increased the risk of all-cause mortality by 45% compared to the normoglycemic group. It emphasizes the additional risk of prediabetes for young patients beyond diabetes, which should be considered earlier in prevention.

There are few studies demonstrating the prevalence and importance of prediabetes in young adults who underwent CAG. Among young STEMI patients, the prevalence of prediabetes was 24% [7]. In addition, the prevalence of prediabetes was significantly higher in young patients receiving CAG compared to the corresponding age group in the general population [3]. Our results indicated that over 1/3 of young patients were characterized as prediabetes, which emphasized the importance of routine testing for prediabetes in young patients undergoing CAG.

It was controversial whether prediabetes increases the risk of cardiovascular disease and all-cause mortality [14–16]. Previous studies have demonstrated the increased risk of CVD and all-cause mortality were associated with the presence of prediabetes in young adults without diabetes and prior CVD [17]. In Japanese workers, the relationship between prediabetes and all-cause mortality has also been investigated [18]. However, an innovative cross-sectional study to reduce cardiovascular complications in diabetes (ARTEMIS) showed that prediabetes is not associated with the risk of cardiovascular mortality and all-cause mortality in patients with CAD [13]. In older adults, prediabetes and newly diagnosed diabetes are not significantly associated with a higher risk of all-cause mortality [19–21]. Huang et al. found no significant association between prediabetes alone and long-term mortality in the general population, but the predictive power of prediabetes for the mortality risk appears to be stronger among low-risk populations (younger and White participants) [22].

Prediabetes was also defined according to various criteria in different guidelines and studies, including impaired glucose tolerance (IGT) and impaired fasting glucose (IFG). IGT is defined as an oral glucose tolerance test 2-h plasma glucose of 7.8–11.0 mmol/L, while IFG is defined by the World Health Organization (WHO) and the ADA as fasting blood glucose of 6.1–6.9 mmol/L (IFG-WHO) and 5.6–6.9 mmol/L (IFG-ADA), respectively [23, 24]. According to Echouffo-Tcheugui’s study, prediabetes in different definitions was related to adverse prognostic risks, including cardiovascular disease, renal disease, and all-cause mortality, with varying effect sizes, depending on the definitions used [23]. Warren’s study reported that HbA1c was more specific than postprandial glucose in screening for prediabetes, improving risk discrimination for clinical complications [25]. On the other hand. a large cross-sectional study has shown that prediabetes is associated with worse outcomes, regardless of the definition adopted [26]. Huang et al. have revealed through meta-analysis that prediabetes was associated with an increased risk of adverse events regardless of different definitions both in the general population and patients with atherosclerotic cardiovascular disease [9, 27]. Nonetheless, further studies are warranted to verify whether the definition of prediabetes would affect the prognosis in the large-scale cohort study of young patients.

Our study showed that prediabetes and diabetes detected by HbA1c can predict a higher risk of all-cause mortality in young adults undergoing CAG. However, in the subgroup analysis, prediabetes and diabetes increased the risk of long-term mortality mainly in young patients received CAG without comorbidities at baseline and the association was insignificant in those patients with AMI, CAD, CHF, CKD or undergoing PCI at baseline. This may partially explain the controversy over prediabetes as a risk factor for all-cause mortality. In patients with existing comorbidities or older age, who have higher mortality and complex risk factors, the role of type 2 diabetes and prediabetes may be masked. However, in younger adults with fewer risk factors, prediabetes and type 2 diabetes may turn out to be one of the primary risk factors. In this study, we confirmed that in young patients undergoing CAG, prediabetes still appeared to be significantly associated with all-cause mortality. Therefore, prediabetes may remain a concern for young patients undergoing CAG.

## Limitation

Several limitations exist in the study. First, as an observational study, our results were influenced by its nature and do not reflect direct cause-and-effect relationships. However, we included more patients compared with previous studies [28–30] and our results still provided a reference for the debates on the prognostic impact of prediabetes. Second, although we have made adjustments for variables as much as we can, there could be potential confounding factors that we may have overlooked. We were unable to evaluate data from additional aspects since our data did not contain concrete causes of mortality and other adverse events. Third, we failed to investigate the evolution in glucose metabolism over time and we did not have sufficient data on fasting glycemia to evaluate the effect of prediabetes on mortality in various definitions. The possibility that the increase in mortality resulted from the conversion of prediabetes to diabetes cannot be excluded. Further study on tracking the changes of prediabetes in young patients receiving CAG is recommended.

## Conclusion

In summary, our study demonstrated that prediabetes was common among young patients undergoing CAG and prediabetes was an independent risk factor for all-cause mortality among them, especially in patients without previous complications.

## Data Availability

The datasets generated and analyzed during the current study are not publicly available due to the institution policy but are available from the corresponding author on reasonable request.

## Abbreviations

ACEI: Angiotensin-converting enzyme inhibitor
ARB: Angiotensin receptor blocker
ADA: American Diabetes Association
AMI: Acute myocardial infarction
CAG: Coronary artery angiography
CAD: Coronary artery disease
CKD: Chronic kidney disease
CHF: Congestive heart failure
ECMS: Electronic clinical management system
eGFR: Estimated glomerular filtration rate
HT: Hypertension
LDL-C: Low-density lipoprotein cholesterol
PCI: Percutaneous coronary intervention
STEMI: ST-elevation myocardial infarction
TC: Total cholesterol
TG: Triglycerides
