# Correction: Prediabetes and all-cause mortality in young patients undergoing coronary artery angiography: a multicenter cohort study in China

**DOI:** 10.1186/s12933-023-01902-8

**Published:** 2023-06-29

**Authors:** Yibo He, Hongyu Lu, Yihang Ling, Jin Liu, Sijia Yu, Ziyou Zhou, Tian Chang, Yong Liu, Shiqun Chen, Jiyan Chen

**Affiliations:** 1 Department of Cardiology, Guangdong Cardiovascular Institute, Guangdong Provincial People’s Hospital, Guangdong Academy of Medical Sciences, Guangzhou, 510080 China; 2 Department of Guangdong Provincial Key Laboratory of Coronary Heart Disease Prevention, Guangdong Cardiovascular Institute, Guangdong Provincial People’s Hospital, Guangdong Academy of Medical Sciences, Southern Medical University, Guangzhou, 510080 China; 3 The Second School of Clinical Medicine, Southern Medical University, Guangzhou, 510515 Guangdong China; 4 School of Medicine, South China University of Technology, Guangzhou, 510006 China; 5 Global Health Research Center, Guangdong Provincial People’s Hospital, Guangdong Academy of Medical Science, Southern Medical University, Guangzhou, 510100 China

**Correction: Cardiovascular Diabetology (2023) 22:42** 10.1186/s12933-023-01776-w

After the publication of the original article [1], it came to the authors’ attention that a piece of funding information for the study presented has been inadvertently omitted. The section on Funding should therefore have read as follows:

“This work was supported by grants from Guangdong Provincial science and technology project (2020B1111170011); Guangdong Provincial science and technology project (KJ022021049); Guangdong Provincial Key Laboratory of Coronary Heart Disease Prevention (No.2017B030314041; Y0120220151), National Science Foundation for Young Scientist of China (Grant No.82070360) and High-level Hospital Construction Project (DFJH2020026). The work was not funded by any industry sponsors. The study sponsor/funder was not involved in the design of the study; the collection, analysis, and interpretation of data; writing the report; and did not impose any restrictions regarding the publication of the report.”

## References

[1] He Y, Lu H, Ling Y, Liu J, Yu S, Zhou Z, Chang T, Liu Y, Chen S, Chen J (2023). Prediabetes and all-cause mortality in young patients undergoing coronary artery angiography: a multicenter cohort study in China. Cardiovasc Diabetol.

